# Pronounced genetic differentiation and recent secondary contact in the mangrove tree *Lumnitzera racemosa* revealed by population genomic analyses

**DOI:** 10.1038/srep29486

**Published:** 2016-07-06

**Authors:** Jianfang Li, Yuchen Yang, Qipian Chen, Lu Fang, Ziwen He, Wuxia Guo, Sitan Qiao, Zhengzhen Wang, Miaomiao Guo, Cairong Zhong, Renchao Zhou, Suhua Shi

**Affiliations:** 1 State Key Laboratory of Biocontrol and Guangdong Provincial Key Laboratory of Plant Resources, Sun Yat-sen University, Guangzhou 510275, China; 2 Hainan Dongzhai Harbour National Nature Reserve, Haikou 571129, China

## Abstract

Systematically investigating the impacts of Pleistocene sea-level fluctuations on mangrove plants may provide a better understanding of their demographic history and useful information for their conservation. Therefore, we conducted population genomic analyses of 88 nuclear genes to explore the population dynamics of a mangrove tree *Lumnitzera racemosa* across the Indo-West Pacific region. Our results revealed pronounced genetic differentiation in this species between the populations from the Indian Ocean and the Pacific Ocean, which may be attributable to the long-term isolation between the western and eastern coasts of the Malay Peninsula during sea-level drops in the Pleistocene glacial periods. The mixing of haplotypes from the two highly divergent groups was identified in a Cambodian population at almost all 88 nuclear genes, suggesting genetic admixture of the two lineages at the boundary region. Similar genetic admixture was also found in other populations from Southeast Asia based on the Bayesian clustering analysis of six nuclear genes, which suggests extensive and recent secondary contact of the two divergent lineages in Southeast Asia. Computer simulations indicated substantial migration from the Indian Ocean towards the South China Sea, which likely results in the genetic admixture in Southeast Asia.

The climatic oscillations that characterized the Pleistocene glaciations have deeply affected the geographical distribution and evolutionary trajectory of plants on the earth^1^,^2^. Although ice sheets did not cover the tropics during the glaciations, sea level changes, as the extending and retreating of ice sheets, have clearly influenced the population dynamics of tropical plants, especially coastal plants^2^,^3^,^4^. Mangroves are one of the most important plant communities growing in the intertidal zones of tropical and subtropical coasts and play a significant role in coastal ecosystems^5^,^6^. They affect carbon balancing, offer protection from tsunamis and hurricanes, and affect fishery production^6^. Dispersed mainly by seawater, propagules of mangrove plants are generally considered to have the ability of long-distance dispersal (LDD)^7^. However, the potential of LDD was found to be limited because of many factors including land barriers and ocean circulations^8^,^9^. In the Indo-West Pacific (IWP) region, the Malay Peninsula plays a major role in promoting the genetic differentiation between populations from the eastern and western coasts^10^. During the glacial periods, falls in sea levels caused the Malay Peninsula, Sumatra and Java to become connected and form the Sunda Land, which halted the exchange of seawater between the Indian Ocean and the South China Sea (SCS)^11^,^12^. This glacial isolation resulted in genetic divergence between populations from these two oceans, which has recently been observed in many mangrove species^10^,^13^,^14^,^15^,^16^,^17^,^18^,^19^,^20^,^21^,^22^.

The habitats of mangrove plants during the glacial periods would be submerged by the rising sea levels in the interglacial periods, and mangrove plants had to move with the shifting of the coastline^23^. Thus the rising sea levels provided opportunities for secondary contact of the isolated populations, especially at the boundary regions^24^. This phenomenon has been described in previous studies on mangrove species^9^,^16^,^25^; however, such genetic admixture remains to be confirmed due to two major reasons. First, those studies could not exclude ancestral polymorphism as a possible explanation^10^ because too few markers were used or low genetic differentiation was observed. Second, other recent studies highlighted the blocking role of contemporary ocean currents in maintaining the genetic breaks between different oceans in *Rhizophora* species based on microsatellite markers and simulations of ocean circulation patterns^26^,^27^,^28^. Such genetic discontinuity may be a species-specific phenomenon because the dispersal ability of different mangrove species can vary significantly^7^. Therefore, the genetic admixture has not been thoroughly explained for mangrove plants, and a more precise investigation is still needed.

*Lumnitzera* (Combretaceae), a genus consisting of two species *L. racemosa* and *L. littorea*, is an important component of mangrove forests in the IWP region^29^. *L. racemosa* is widely distributed from East Africa through Indo-Malesia to Tonga^29^,^30^. It usually grows in the high intertidal zones towards the inland within the estuary system. *L. racemosa* produces tiny fruits with a fibrous portion in the fruit wall. The seeds are protected by a hard layer of sclerenchyma tissue inside the outer corky layer of the fruit wall and could generally keep in seawater for more than 20 days and disperse well with ocean currents^29^,^31^. A previous study revealed low within-population genetic diversity and strong genetic structure between the Indian Ocean and the Pacific Ocean in *L. racemosa* based on inter simple sequence repeat (ISSR) markers^19^. However, no analysis of genetic admixture was conducted, even though substantial gene flow is expected to have occurred in this species, given its high dispersal capability.

In this study, we used population genomic analyses to assess the impacts of sea level oscillations and ocean currents on the population history of *L. racemosa* in the IWP region. 88 nuclear genes were chosen regardless of their functions and sequenced for five pooled population samples using the Illumina platforms, a cost-effective and efficient method in population allele frequency estimation, to obtain the genetic structure of *L. racemosa* at the genomic level. Because Illumina sequencing is based on pooled population samples, population we used for Illumina sequencing and analysis in this paper is different from the conventional “population” concept. Six of the 88 genes from 12 additional populations were sequenced by the Sanger method to assess the general pattern of genetic diversity in the IWP region. We used IMa2 analysis to estimate the splitting time, population size and pairwise migration rate parameters between populations from different regions. Based on these results, we sought to determine whether 1) glacial sea-level drops caused the genetic divergence between populations from the eastern and western coasts of the Malay Peninsula and 2) genetic exchange occurred from secondary contacts of previously isolated populations of *L. racemosa* after rising sea levels. A better understanding of these questions will shed light on the evolutionary history of *L. racemosa* and provide useful information for the conservation of mangroves and other coastal communities that face the threat of rising sea levels under global warming.

## Results

### Genetic diversity

Using the Illumina platforms, a total of 11.45 to 14.53 million 75 (QH and SY) or 90 bp pair-end reads (KP, JH and KD) were generated and aligned to 84 to 88 genes with mean depth ranging from 5127× to 6061× (Tables 1 and 2; see Methods for details). A total of 26 to 724 single nucleotide polymorphism (SNP) sites were detected from 12 to 78 polymorphic genes in five populations (Table 2). The nucleotide diversity (π) for each gene from the five populations and the corrected nucleotide polymorphism (θ) within each population were calculated to assess the genetic diversity. According to these results, the two populations from China harboured the lowest level of genetic diversity (mean nucleotide diversity 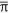 = 0.080 × 10^−3^ and θ = 0.077 × 10^−3^ in QH; 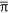 = 0.112 × 10^−3^ and θ = 0.080 × 10^−3^ in SY). The two populations from Malaysia (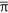 = 0.156 × 10^−3^ and θ = 0.133 × 10^−3^ in JH; 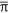 = 0.099 × 10^−3^ and θ = 0.084 × 10^−3^ in KD) had slightly higher polymorphism than those from China. The Cambodian population (KP) showed the highest level of genetic diversity (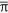 = 1.021 × 10^−3^ and θ = 2.434 × 10^−3^), which was an order of magnitude higher than those in other populations. Relative to the diversity at the population level, *L. racemosa* showed a higher level of genetic diversity at the species level (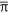 = 2.833 × 10^−3^ and θ = 2.291 × 10^−3^).

**Figure 1:**
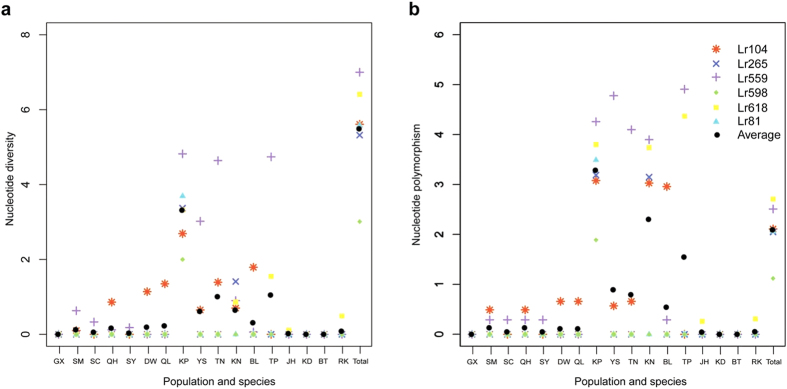
Statistics of genetic variation based on Sanger sequencing data. (**a**) Nucleotide diversity π and (**b**) Nucleotide polymorphism θ at six nuclear genes in 17 populations of *Lumnitzera racemosa* from the Indo-West Pacific region. Each symbol represents one of the six genes and the average value of six genes from each population. Population abbreviations are defined in Table 1.

Furthermore, we sequenced six of the 88 nuclear genes (Lr104, Lr265, Lr559, Lr598, Lr618 and Lr81) from 295 individuals in 17 populations (including five populations used in the Illumina sequencing) across the IWP region using the Sanger method. At the species level, π ranged from 3.010 × 10^−3^ to 7.000 × 10^−3^ with an average of 5.488 × 10^−3^ and θ ranged from 1.120 × 10^−3^ to 2.710 × 10^−3^ with an average of 2.093 × 10^−3^. At the population level, populations from China (GX, SM, SC, QH and SY), Australia (DW and QL), Malaysia (JH and KD) and Sri Lanka (BT and RK) had no or extremely low levels of polymorphism; however, populations from Cambodia (KP), Thailand (YS, TN and KN), Indonesia (BL) and Singapore (TP) harboured relatively high levels of polymorphism (Fig. 1; Supplementary Table S1). These results are consistent with those obtained from Illumina sequencing.

### Haplotype networks and distributions

**Figure 2:**
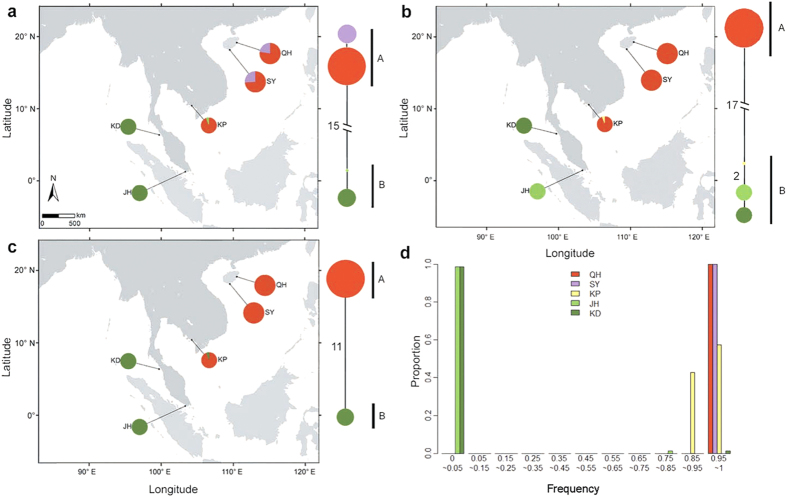
Haplotype information of *Lumnitzera racemosa* in the five populations based on Illumina sequencing data. (**a–c**) Show the haplotype networks and distributions of three polymorphic gene segments (Lr191, Lr546b and Lr667a). The haplotype distributions were created by GenGIS version 2.4.0^47^ and MapMaker version 1.0 (http://kiwi.cs.dal.ca/GenGIS/MapMaker)^48^. Each colour represents a single haplotype. The circle sizes in the geographic distributions and the haplotype networks are proportional to the sample size of each population and the frequency of each haplotype, respectively. Each branch with more than one mutational step is labelled. Capital letters A and B beside the haplotype networks represent group A and B, respectively. (**d**) The frequency histogram of group A in 75 highly divergent gene segments from the five populations. Group A is defined based on the criteria described in the Materials and Methods section. Population abbreviations are defined in Table 1.

Using the linkage information of SNPs in each pair of reads, the haplotypes were inferred by an Expectation-Maximization algorithm. When the distance between two proximal SNPs at some genes was longer than the covered length of paired reads, these genes were cut into two segments for haplotype inference. Finally, we constructed haplotype networks for 88 polymorphic segments from 85 polymorphic genes across the five populations (Fig. 2a–c; Supplementary Fig. S2). For most segments, the networks revealed two highly divergent groups. By hierarchical cluster analysis of complete linkage method (see Methods for details), the haplotypes of 75 gene segments were clustered into two groups (hereafter referred to as group A and group B). The two populations from China (QH and SY) had haplotypes from group A, while the two populations from the western coasts of the Malay Peninsula (JH and KD) had haplotypes from group B. There were almost no intermediate haplotypes between the two divergent groups in these haplotype networks. This result indicated pronounced genetic divergence between the populations from the Pacific Ocean and the Indian Ocean. Notably, the haplotypes of these two groups were both found within the Cambodian population (KP) in 69 of the 75 segments. The frequency spectrum of the haplotypes of group A was calculated for each of the five populations (Fig. 2d). Consistent with haplotype networks, the two populations QH and SY had the haplotypes of group A with a frequency of only 1 in all the 75 segments, while JH or KD had almost no haplotypes in this group except for one segment. In the KP population, 85–99% of the genetic composition in the 69 segments came from group A, while the remaining 1–15% of the genetic composition belonged to group B.

**Figure 3:**
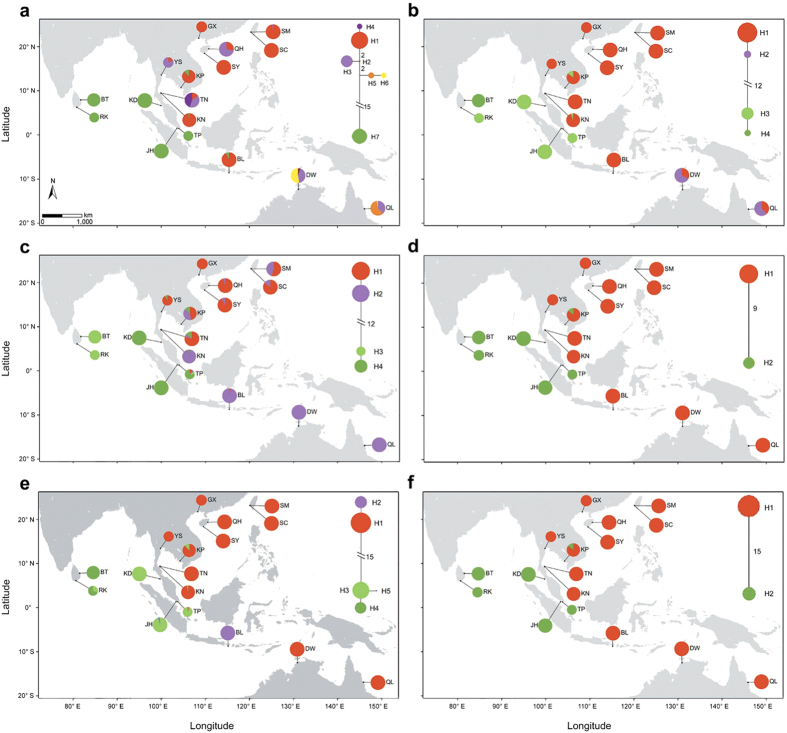
Haplotype networks and distributions of six nuclear genes across 17 populations of *Lumnitzera racemosa* in the Indo-West Pacific region. (**a**) Lr104, (**b**) Lr265, (**c**) Lr559, (**d**) Lr598, (**e**) Lr618 and (**f**) Lr81. The haplotype distributions were created by GenGIS version 2.4.0^47^ and MapMaker version 1.0 (http://kiwi.cs.dal.ca/GenGIS/MapMaker)^48^. Each colour represents a single haplotype. The circle sizes in the geographic distributions and the haplotype networks are proportional to the sample size of each population and the frequency of each haplotype, respectively. Each branch with more than one mutational step is labelled. Population abbreviations are defined in Table 1.

We also constructed haplotype networks of six nuclear genes from 17 populations across the IWP region using Sanger data (Fig. 3). Again, two highly divergent groups were separated by 9–15 mutational steps for these genes with an aligned length of 803–1432 bp. Similar to the results from Illumina sequencing, one group dominated the populations from China (GX, SM, SC, QH and SY) and Australia (DW and QL) while the other group was detected mainly in the populations from Malaysia (JH and KD) and Sri Lanka (BT and RK). The haplotypes from both groups were observed within populations from Southeast Asia, including Cambodia (KP), Thailand (YS, TN and KN), Indonesia (BL) and Singapore (TP). The mixing proportions of the two groups varied among different genes and different populations.

### Genetic differentiation and population structure estimation

**Figure 4:**
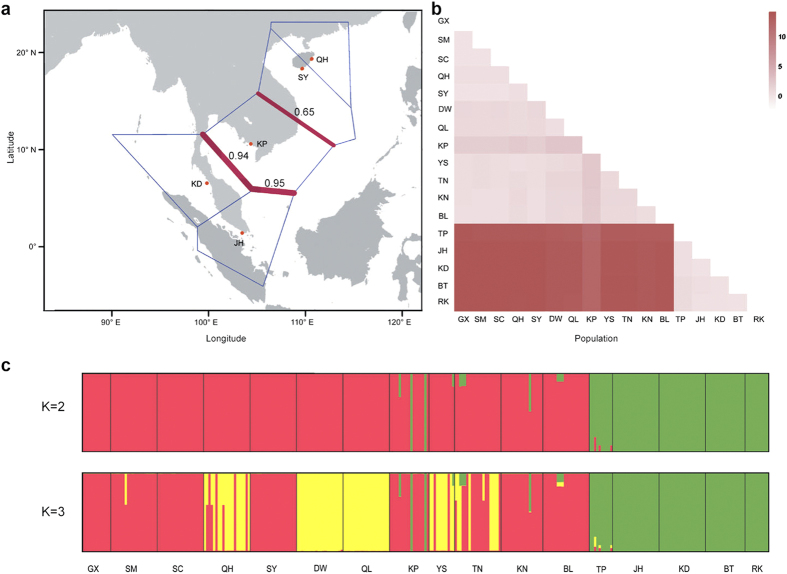
Genetic differentiation and population structure across populations of *Lumnitzera racemosa* in the Indo-West Pacific region. (**a**) Results of the BARRIER analysis based on 85 F_ST_ matrices obtained from Illumina sequencing data. The digital map of this area was created by MapMaker version 1.0 (http://kiwi.cs.dal.ca/GenGIS/MapMaker)^48^. The blue line represents a Voronoï tessellation. The red line represents detected barriers and the thickness corresponds to the strength. The number label beside the red line indicates the percentage of genes that support this barrier. (**b**) A heatmap of the averages of pairwise Kxy values of six nuclear genes among 17 populations. The colour depth is proportional to the level of genetic differentiation. (**c**) The results of the Bayesian clustering analysis for 295 *Lumnitzera racemosa* individuals based on six nuclear genes. Each bold bar separated by a black line represents one population and each colour represents one cluster. The best clustering number was estimated to be equal to 2 (K = 2).

The F-statistic (F_ST_) for each gene segment was calculated to reveal the levels of genetic differentiation between populations. The averages of pairwise F_ST_ values (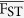) among the five populations were presented in Supplementary Table S2. Of the 88 polymorphic segments, a relatively low level of population differentiation was identified within each of the two regions, China and western coast of the Malay Peninsula (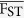 = 0.031 between QH and SY, 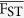 = 0.156 between JH and KD). However, there were very high levels of differentiation between these two regions (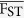 range from 0.901 to 0.923). Consistent with the result of the haplotype frequency spectrum, the Cambodian population (KP) was more closely related to the two Chinese populations (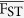 = 0.065 and 0.044) than to the two Malaysian populations (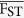 = 0.847 and 0.846). Based on 85 matrices of pairwise F_ST_ among the five populations, BARRIER analysis revealed that the strongest genetic barrier existed between the populations from the Pacific Ocean and the Indian Ocean, which was supported by 94% of the genes. Another genetic barrier was identified between Cambodia and China, with 65% of genes (Fig. 4a).

A similar conclusion can be drawn from the pairwise nucleotide divergence (Kxy) of the six genes sequenced by the Sanger method (Fig. 4b; Supplementary Tables S3–8). The strongest genetic differentiation was between the populations from the Indian Ocean (Malaysia and Sri Lanka) and the Pacific Ocean (China and Australia). Within the Pacific Ocean, the Kxy values suggested a divergence between the populations from China and Australia. The lowest pairwise Kxy values were detected among populations within each of the Indian Ocean, China and Australia regions. By comparison, the patterns of genetic differentiation were much more complex in the populations from Southeast Asia. Because of the mixing of the two divergent haplotype groups in different proportions as mentioned above, most of the Southeast Asia populations (KP, YS, TN, KN and BL) were closely related to populations from China and Australia, whereas the population from Singapore (TP) was closely related to the Indian Ocean populations.

The Bayesian clustering algorithm-based STRUCTURE analysis revealed that the optimum K value was 2 when using the Sanger dataset of six nuclear genes from 295 individuals in 17 populations (Fig. 4c; Supplementary Fig. S3). For K = 2, the populations from China (GX, SM, SC, QH and SY) and Australia (DW and QL) were assigned to one cluster, and the populations from Malaysia (JH and KD) and Sri Lanka (BT and RK) were assigned to the other one. This result corresponds to the divergence between the populations from the Indian Ocean and the Pacific Ocean. In particular, those two clusters co-occurred in the populations from Cambodia (KP), Thailand (YS, TN and KN), Indonesia (BL) and Singapore (TP). Except for TP, approximately 86.5% to 98.5% of the genetic components came from the dominant cluster in the Pacific Ocean, while the rest came from the Indian Ocean lineage. By contrast, approximately 3.4% of the genetic components of TP came from the Pacific Ocean lineage and the remainder came from the Indian Ocean lineage. These results provide a strong signal of genetic admixture of the two divergent lineages in the populations from Southeast Asia. When K = 3, the populations from China and Australia were subdivided into two clusters, which agrees with the result of the Kxy statistics.

### Demographic history analysis

**Figure 5:**
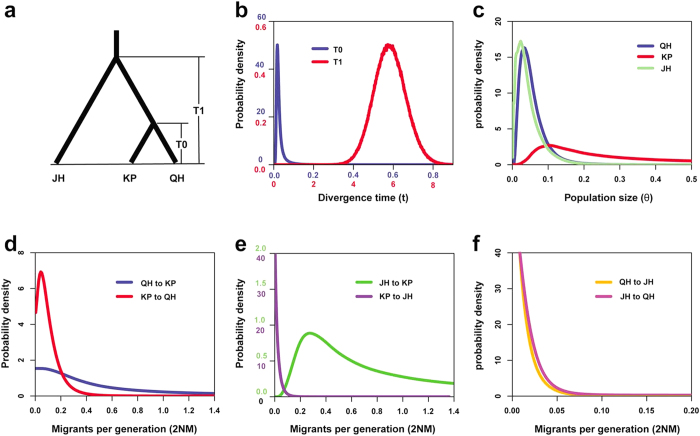
Probability density plots of the demographic parameters estimated under the isolation-with-migration model for populations from China (QH), Cambodia (KP) and Malaysia (JH). (**a**) A schematic of isolation with migration model. T0 represents the divergence time between the QH and KP populations. T1 represents the divergence time between JH and the most recent common ancestor of QH and KP. (**b**) Probability density estimation of the divergence times. (**c**) Probability density estimation of the population sizes for the three populations. (**d–f**) Probability density estimation of pairwise directional migration rates (2NM). Population abbreviations are defined in Table 1. Note that different scales were used for the horizontal and vertical coordinates in B and E with different colours.

We performed the IMa2 simulation runs to infer the population history of *L. racemosa* across the IWP region under the isolation-with-migration (IM) model based on six nuclear genes from Sanger sequencing. Three populations from China (QH), western Malaysia (JH) and Cambodia (KP) were selected to represent populations from the Pacific Ocean, the Indian Ocean and the Indian-Pacific boundary, respectively (Fig. 5a). The effective sample size (ESS) values for the estimated parameters all exceeded 100, indicating sufficient Markov chain Monte Carlo (MCMC) mixing across parameter space for reasonable estimates of the parameters^32^. The plots of the marginal posterior probability densities of the demography parameters were shown in Fig. 5. We used an assumed mutation rate ranging from 3.0 to 9.0 × 10^−9^ substitutions per site per year (s/s/y) based on the neutral mutation rate of 6.1 × 10^−9^ in vascular plants^33^. The divergence time between QH and KP was 0.019 (95% of the highest posterior density intervals (HPDI): 0.007–0.077) in units of mutation time scale (T = tu). This corresponds to 0.006 million years ago (Mya) under the mutation rates of 3.0 × 10^−9^ s/s/y, and 0.002 Mya under 9.0 × 10^−9^ s/s/y, respectively. The estimated divergence time between JH and the most recent common ancestor (MRCA) of QH and KP was 5.729 (95% HPDI: 4.271–7.430), corresponding to 0.650–1.949 Mya under different mutation rates. The effective population sizes of QH, KP and JH were estimated to be 0.033 (95% HPDI: 0.007–0.129), 0.105 (95% HPDI: 0.023–1.709) and 0.023 (95% HPDI: 0.003–0.119) in units of θ. All the estimated migration rates were very low. Based on the likelihood ratio (LLR) test, only gene flow from JH to KP, among all the pairwise comparisons, was statistically greater than zero (2NM_JH −> KP_ = 0.276, 95% HPDI: 0.052–5.769, LLR = 140.760, P < 0.001), and gene flow in this direction was two orders of magnitude higher than in the opposing direction (KP to JH, 2NM_KP −> JH_ = 0.001). Moreover, the numbers of bidirectional migrants per generation between QH and KP (2NM_QH −> KP_ = 0.004 and 2NM_KP −> QH_ = 0.043) were higher than those between QH and JH (2NM_QH −>JH_ = 0.0002 and 2NM_JH −> QH_ = 0.0007).

## Discussion

The objective of this study was to use population genomic analyses to investigate the genetic diversity and genetic exchange in populations of *L. racemosa* in the IWP region. In most of the 88 genes, strong genetic differentiation is identified between populations from the Indian Ocean and the Pacific Ocean, suggesting long-term glacial isolation between these two oceanic regions due to steep sea-level drops. It should be noted that these two lineages co-occurred within populations from the coasts of Southeast Asia, which provides compelling evidence for secondary contact of the two divergent lineages. Moreover, re-colonization processes after the last glaciation should be the major reason for the secondary contact at the boundary region. Our study provides new insights into the population dynamics of *L. racemosa* and enriches our current understanding about the evolutionary history of mangrove plants.

The haplotype networks of 88 genes across the IWP region revealed that populations from the Indian Ocean and the Pacific Ocean fell into two highly divergent groups (Figs 2 and 3; Supplementary Fig. S2). This genealogical break was further supported by Bayesian clustering (Fig. 4c) and the statistics of genetic differentiation (F_ST_ and Kxy) (Fig. 4b, Supplementary Tables S2–8). Our BARRIER analysis suggested that the barrier laid along the Malay Peninsula, as a part of the well-known land barrier Sunda Land, might be the major reason for the genetic discontinuity of two oceanic regions in *L. racemosa* (Fig. 4a). During the Pleistocene glaciations, the Sunda Land cut off the exchange of seawater between the Indian Ocean and the SCS^11^ and separated *L. racemosa* into two isolated regions. Similar east-west differentiation across the Malay Peninsula has also been observed in this species and its sister species using ISSR markers^19^,^20^. It has also been observed in other mangrove species including *Rhizophora* species^10^, *Ceriops* species^13^,^14^,^15^,^16^,^17^, *Bruguiera gymnorrhiza*^18^,^21^ and *Excoecaria agallocha*^22^. IMa2 analysis indicated that the divergence between populations from the Indian Ocean and the Pacific Ocean may have occurred 0.650–1.949 Mya under the assumed mutation rates, which corresponds to the Early-Middle Pleistocene. The Pleistocene was characterized by repeated falls and rises of sea levels during the alternating glacial and interglacial periods of the past two million years^2^. Based on our estimation, the genetic division of *L. racemosa* between the Indian Ocean and the Pacific Ocean is likely to be a result of multiple glaciations rather than the last glaciation alone.

Compared to the high divergence between populations from the Indian Ocean and the Pacific Ocean, the populations from China and Australia are fairly closely related, which is consistent with a previous study in *L. racemosa*^19^. Bayesian clustering assigned the individuals from the two regions into the same cluster under the optimum clustering (K = 2), but separated them into two clusters when K = 3 (Fig. 4c). The slight divergence between the populations from the two regions was recovered by the statistics of Kxy (Fig. 4b, Supplementary Tables S3–8). Although the Indonesia Archipelago plays an important role in the genetic differentiation between China and Australia, the gene exchange mediated by the Indonesian through-flow (ITF) current might not be trivial for mangroves. The ITF moves seawater from the Celebes Sea through the Makassar Strait to the Timor Sea^34^ and may have acted as a major corridor for southward sea-drifted gene flow during the Pleistocene glaciations.

Our study identified apparent haplotype mixing in the populations from Southeast Asia, at the Indo-Pacific boundary. In the Cambodian population (KP), almost all genes showed a combination of the two highly divergent groups (Fig. 2; Supplementary Fig. S2). Similar haplotype mixing was also observed in some other populations from Southeast Asia based on the haplotype networks of six nuclear genes (Fig. 3). Bayesian clustering indicated that 3.4% to 98.5% of the genetic components of the populations from Southeast Asia came from the Pacific Ocean lineage, while the remainder was attributable to the Indian Ocean lineage (Fig. 4c). This pattern may be a result of the retention of ancestral polymorphisms or the result of secondary contact.

Some previous studies about mangroves identified the presence of identical chlorotypes in different populations^9^,^16^,^25^, which is best explained by ancestral polymorphisms^10^. However, in *L. racemosa*, the haplotype networks were dichotomous with missing intermediate haplotypes between the two distinctly common haplotype groups. Although disruptive selection or balancing selection could generate two highly divergent groups without intermediate haplotypes within one population, it is unlikely that almost all 88 randomly chosen genes throughout genome are under the selection effect, suggesting that the pattern observed in *L. racemosa* should not be attributed to the retention of ancestral polymorphisms. Rather, secondary contact of two oceanic lineages from the Indian Ocean and the Pacific Ocean is the most plausible explanation for the haplotype mixing in the populations from Southeast Asia. As described before, the fruits of *L. racemosa* are very tiny and light (<1 cm and ~0.1 g) and they usually do not anchor immediately around the parent trees. Instead, they flow with seawater and wait to colonize until coming into suitable sediment. This could facilitate long-distance dispersal of the seeds of *L. racemosa* by sea currents and promote the successful genetic exchange between populations from different regions. We also observed that several populations in Southeast Asia exhibited an indication of genetic admixture barely in part of the six genes. The discrepancy between the different genes may be attributable to strong genetic drift after secondary contact. Small effective population sizes are expected for these populations in Southeast Asia, which were sometimes sparse and fragmented following sea level changes. After initial mixing, alleles from one lineage might have been randomly and quickly fixed within some genes in these small populations due to strong genetic drift^35^.

Moreover, the IMa2 analysis suggests that the dispersal of *L. racemosa* appears to be asymmetrical between the Indian Ocean and the Pacific Ocean. The estimated gene flow from the western Malaysian population (JH) to the Cambodian population (KP) was two orders of magnitude higher than in the opposite direction (Fig. 5e). The surface currents from the Indian Ocean north-eastward to the SCS are driven by the seasonal monsoons from June to September, while they are driven south-westward from the SCS to the Indian Ocean from November to February^11^,^12^. In our field survey, mature seeds of *L. racemosa* can be observed mainly from July to August and their presence is synchronized with the summer sea current, which might facilitate seed dispersal from the Indian Ocean into the SCS. It can account for the substantial and asymmetrical gene flow between the two oceanic regions in *L. racemosa*. The seasonal ocean currents may also contribute to the genetic homogeneity between the populations from China and Australia.

The secondary contact of two distinct lineages in *L. racemosa* probably occurred recently. As mentioned above, the current KP population was estimated to be formed 0.002–0.006 Mya. This estimate provides a putative upper bound for the mixing of the two lineages. On the other hand, the absence of intermediate haplotypes suggested that there were few intragenic recombinations between the haplotypes of the two groups. Thus, it is likely that the interregional migration detected in *L. racemosa* occurred after the last glaciation since approximately 0.015 Mya^36^, suggesting that substantial gene flow after the last glaciation likely played a major role in the genetic admixture in this species.

Our investigation of the demographic history of *L. racemosa* provides practical information for the conservation of mangrove communities. Because of global warming, the sea level is projected to rise by approximately 1 metre by the end of this century^37^, and extant mangrove populations have to colonize new habitats. To effectively conserve the mangroves as well as other coastal communities, the existing mangrove populations in Southeast Asia should be protected as a priority. On one hand, population admixture was detected mainly in these areas, and this admixture may facilitate the adaptation of mangroves to rapidly changing climates and environments by efficiently increasing the with-population polymorphisms^38^. On the other hand, mangrove forests in Southeast Asia have experienced the most severe disturbance and destruction over the past several decades, such as over-harvesting, aquaculture development and industrial activity^39^.

In summary, our study identified two divergent lineages in *L. racemosa* from the Indian Ocean and the Pacific Ocean, respectively, due to geographical isolations during the Pleistocene glaciations. Interestingly, we provided convincing evidence for recent secondary contact in Southeast Asia after the last glaciation in *L. racemosa*, which highlights the effect of sea-drifted gene flow on the population dynamics of mangrove species. Our findings not only shed light on the influence of the sea level oscillations on the demographic history of mangrove plants over the past two million years but also provide useful information for the conservation of coastal communities.

## Methods

### Plant materials

We sampled two populations of *L. racemosa* from Qionghai (QH) and Sanya (SY) in China, one population from Kep (KP) in Cambodia and two populations from Johor (JH) and Kedah (KD) in Malaysia for Illumina sequencing. Leaves of 17 to 100 individuals each from the five populations were collected for DNA isolation (Table 1). We also sampled a total of 295 individuals of *L. racemosa* from 17 populations (including the five populations mentioned above) from China, Australia, Cambodia, Thailand, Indonesia, Singapore, Malaysia and Sri Lanka for Sanger sequencing (Table 1). For each population, we sampled 10 to 20 individuals. Fresh leaves were stored with silica gel in zip-lock plastic bags for DNA isolation.

### Illumina sequencing

To assess the genetic differentiation and secondary contact in *L. racemosa*, we employed Illumina sequencing for pooled population samples. Different from Zhou, *et al*.^40^, which used dual platforms to make an accurate identification of low-frequency SNPs, we used only the Illumina platform in this study because few within-population false positive SNPs should have little influence on the divergence pattern of *L. racemosa*, given strong among-population genetic differentiation and low within-population polymorphism in *L. racemosa*^19^. Furthermore, the previous study was carried out at the infancy of next generation sequencing (NGS) when accuracy was a real issue and double-platform was useful back then, while the sequencing quality has improved performance now. Thus, pooling individuals from each local population for single platform sequencing is a cost-effective and informative strategy meeting our purpose. For each of the five populations, we prepared pooled leaf samples from all the individuals and isolated the total DNA from the mixed sample using the method described by Zhou, *et al*.^40^. A leaf cDNA library of *L. racemosa* was constructed for one individual sampled from Qionghai, China using the Creator^TM^ Smart^TM^ cDNA Library Construction Kit (Clontech, Mountain View, CA, USA) and 200 clones were sequenced from the library. Based on these sequences, we designed primers for the intron-containing genes and used them for PCR amplification. We validated these primers with one individual from the Qionghai, China population (QH). To ensure homology, 88 genes meeting the following three criteria in each case were used for the five pooled population samples: 1) only one single electrophoretic band was amplified, 2) clear sequence was obtained using Sanger sequencing, and 3) two pairs of primers anchoring different locations for each gene produced completely identical sequences in the overlapped region. The sequences of those 88 genes were employed as reference sequences for further short read mapping. We followed the methods of Zhou, *et al*.^40^ for PCR amplification and PCR product purification of the five pooled population samples. Purified PCR products from 84 to 88 genes were pooled in equal quantities to reach a total of 10 μg for sequencing on the Illumina GA (QH and SY) and HiSeq 2000 platforms (KP, JH and KD, Table 2; BGI, Shenzhen, China).

### Data analysis for Illumina sequencing

MAQ version 0.6.8^41^ was used for short read alignment and single nucleotide polymorphism (SNP) sites identification. We mapped the short reads of each population sample to reference sequences separately using the main parameters: -m 0.002 and -e 200. We then displayed the alignment in a ‘pileup’ text format using the parameters: -Q 200 and -q 30. Based on the pileup result of each population, we plotted the mismatch rate for each site across the reads. These curves showed higher mismatch rates in the first and the last several bases, which likely result from sequencing errors (Supplementary Fig. S1a). Thus, we trimmed these bases of the mapped reads. According to the correlation analysis (Supplementary Fig. S1b), the mismatch rates were stable and low enough when the base quality was equal to or greater than 22. For example, at the base quality of 22, the mismatch rate in the QH population, where the reference sequences came from, was only 0.36%. Therefore, we chose the feasible quality cutoff = 22 to ensure the accuracy of identified SNPs. To avoid the error in SNP identification due to homopolymer and insertion/deletion regions, we masked all bases located in these regions of each population. Putative SNPs of each population were identified using the following criteria: 1) minimum coverage was 100; and 2) minimum minor allele frequency (MAF) was 0.01 in QH and SY, 0.029 in KP, and 0.025 in JH and KD based on their sample sizes. We estimated nucleotide polymorphism θ^42^ using the method of He, *et al*.^43^ to correct the high error rate of Illumina sequencing compared with Sanger sequencing^44^.

Using the linkage information of SNPs in each pair of reads, we inferred haplotypes and their frequencies in each population using an Expectation-Maximization algorithm^45^,^46^. We excluded those haplotypes whose frequencies were less than the MAF of each population and could stem from sequencing errors. The reads generated in this study were 75 or 90 bp pair-end with insert length of 200 bp. Thus, for some genes, if the distance between two proximal SNPs was longer than the covered length of pair-end reads of 350 (200 + 75 × 2) or 380 (200 + 90 × 2) bp, those genes would be cut into two segments to infer haplotypes. Based on the haplotype profiles, we estimated the nucleotide diversity π (the average number of nucleotide differences per site between two sequences) for each gene from each of the five populations. Then, to assess the nucleotide diversity of the species, the weighted average of those values was calculated based on different sample sizes. We used F-statistics (F_ST_) to measure the levels of genetic differentiation between populations. GenGIS version 2.4.0^47^ was used to integrate the haplotype distributions of three selected genes with the geospatial data to visually display the geographic distribution of haplotypes (Fig. 2a–c). The digital map of the area interested was created by MapMaker version 1.0 (http://kiwi.cs.dal.ca/GenGIS/MapMaker)^48^.

We found that haplotypes at almost all gene segments fell into two divergent groups. For each segment successfully sequenced in all five populations and meeting one of the two criteria: 1) containing more than two SNPs or 2) containing two SNPs but having only two haplotypes, we employed the hierarchical cluster analysis of complete linkage method^49^ for haplotype clustering in R software (version 3.13, https://www.r-project.org), in which each of the haplotypes was initially assigned into one group, and then the closest two groups were joined together at each stage until only two groups were left. The two groups were denoted as group A and B, respectively. The frequency distribution of group A was calculated in each of the five populations (Fig. 2d).

Additionally, the genetic boundaries between the populations were inferred by the Monmonier’s^50^ maximum difference algorithm in the BARRIER software version 2.2^51^ based on a matrix of pairwise F_ST_ values among populations. The Illumina sequencing technology successfully produced 85 matrices of pairwise F_ST_ for all five populations; therefore, we assessed the robustness of the inferred barriers using these data and considered them as reliable if supported by at least half of these genes.

### Sanger sequencing and data analysis

Six of the 88 nuclear genes (Lr104, Lr265, Lr559, Lr598, Lr618 and Lr81) were sequenced by the Sanger method. The sequences of the six nuclear genes were aligned with the ClustalX program^52^ and edited in SeqMan (version 7.10, DNAStar, London, UK). The haplotypes were inferred with PHASE version 2.1.1^53^. Haplotype networks were constructed using the median-joining method^54^ in NETWORK (version 4.5.1.6, Fluxus Technology Ltd). DnaSP version 5.10.00^55^ was used to calculate the polymorphism index (haplotype diversity Hd, nucleotide diversity π, and nucleotide polymorphisms θ) and pairwise nucleotide divergence Kxy (average number of nucleotide differences between populations). Similar to the analysis of Illumina sequencing, the haplotype distributions of each gene were combined with the geographical information using GenGIS version 2.4.0^47^ and MapMaker version 1.0^48^.

The individual-based genetic structure and population admixture were evaluated based on the 93 polymorphic sites of the six nuclear genes by Bayesian clustering using STRUCTURE version 2.3.3^56^. The number of genetic clusters (K) was pre-assigned from 1–10 with 10 replicates per K to test the stability of the results. All the runs used 1.0 × 10^6^ Markov chain Monte Carlo (MCMC) iterations after a burn-in of 200,000 steps and used the admixture model with the assumption of correlated allele frequencies among clusters^57^. The most likely number of clusters K was estimated according to the ΔK statistics method^58^ implemented in Structure Harvester^59^. The final results for the STRUCTURE analysis were visualized using DISTRUCT version 1.1^60^.

### Demographic history analysis

To infer the evolutionary history of *L. racemosa* in the IWP region, the effective population sizes, population divergence times and bi-directional migration rates between populations were estimated under the isolation-with-migration (IM) model using the IMa2 program with the Sanger data of six genes^61^. The populations from the Pacific Ocean, the Indian Ocean and the Indian-Pacific boundary were represented by the three selected populations from China (QH), western Malaysia (JH) and Cambodia (KP), respectively. As identified by the Bayesian clustering and the Kxy statistics, the KP population was more closely related to the QH population than to the JH population. Thus, we employed the prior phylogenetic tree showing that KP and QH shared a more recent common ancestor rather than JH, as shown in Fig. 5a. Because of the uncertain mutation rate of both *L. racemosa* and other plants in the Combretaceae family, we assumed a mutation rate ranging from 3.0 to 9.0 × 10^−9^ substitutions per site per year (s/s/y) based on the neutral mutation rate of 6.1 × 10^−9^ in vascular plants^33^. Because the IMa2 program assumes that all loci are free of recombination, we used the IMgc program to obtain the longest region without four gametic types for each locus^62^. Based on the results of early trial runs, we conducted the simulation for a burn-in of 1.0 × 10^6^ steps and 2.0 × 10^7^ steps with 40 chains under the HKY model. To check for the convergence of the simulations, we performed three independent runs with different seed numbers. The posterior distributions of parameters were estimated using 200,000 genealogies sampled from well-mixed runs and the parameters selected to represent the demography were obtained from the peaks of the posterior distributions and 95% of the highest posterior density intervals (HPDI)^63^. All the demographic parameters were scaled by the geometric mean of the mutation rates across the six genes^32^. A likelihood ratio (LLR) test was used to assess the significance of the migration rate estimates (*α* = 0.001)^63^.

## Additional Information

**Accession codes:** Sequences obtained by Sanger method were deposited in GenBank with accession numbers KF477441–KF477511, KF496922–KF496926, KF553911–KF553922, KJ476444–KJ476454 and KT246108–KT246120. Raw short reads of five population samples were deposited in the NCBI Sequence Read Archive with the accession number of SRP038914 and SRP044209.

**How to cite this article**: Li, J. *et al*. Pronounced genetic differentiation and recent secondary contact in the mangrove tree *Lumnitzera racemosa* revealed by population genomic analyses. *Sci. Rep.* **6**, 29486; doi: 10.1038/srep29486 (2016).

## Supplementary Material

Supplementary Information

## Figures and Tables

**Table 1:** Sampling localities, coordinates and sample sizes of 17 populations of *Lumnitzera racemosa* from the Indo-West Pacific region used in this study.

Population abbreviation	Locality	Latitude	Longitude	Sample size
GX	Fangchenggang, Guangxi, China	21.62°N	108.35°E	12
SM	Shengmumiao, Tainan,Taiwan, China	23.03°N	120.13°E	20
SC	Sicao,Tainan,Taiwan, China	23.02°N	120.11°E	20
QH	Qionghai, Hainan, China	19.23°N	110.61°E	20 (100)
SY	Sanya, Hainan, China	18.23°N	109.63°E	20 (100)
DW	Channel island, Darwin, Australia	12.47°S	130.85°E	20
QL	Cairns Airport, Queensland, Australia	16.87°S	145.75°E	20
KP	Kep, Cambodia	10.49°N	104.33°E	17 (17)
YS	Yi San, Samut Songkhram,Thailand	13.35°N	99.92°E	11
TN	Thong Nian Bay, Thong Nian, Nakhon Si Thammarat, Thailand	9.30°N	99.80°E	20
KN	Khanom, Thong Nian, Nakhon Si Thammarat, Thailand	9.22°N	99.85°E	18
BL	Kuta, Bali,Indonesia	8.72°S	115.19°E	20
TP	Tiong Poh,Singapore	1.29°N	103.85°E	10
JH	Kukup, Johor, Malaysia	1.33°N	103.44°E	20 (20)
KD	Lankawi,Kedah,Malaysia	6.45°N	99.81°E	20 (20)
BT	Batticaloa, Sri Lanka	7.72°N	81.70°E	17
RK	Rekawa, Sri Lanka	6.05°N	80.85°E	10
At species level				295 (257)

The number in the brackets is the sample size for Illumina sequencing.

**Table 2:** Illumina sequencing data and polymorphism statistics for five population samples of *Lumnitzera racemosa*.

	QH	SY	KP	JH	KD
Number of sequenced genes	88	88	86	84	85
Illumina platforms	Illumina GA	Illumina GA	HiSeq 2000	HiSeq 2000	HiSeq 2000
Length of short paired-end reads	75	75	90	90	90
Total reads (×10^6^)	15.18	13.24	12.77	12.82	12.81
Aligned reads (×10^6^)	14.53	12.58	12.03	11.45	11.76
Percentage of mapped reads (%)	95.76	94.98	94.21	89.28	91.83
Mean depth	5990×	5127×	6061×	5617×	5618×
Number of polymorphic genes	29	31	78	12	14
Number of SNPs	36	39	724	38	26
Average of π (×10^−3^)	0.080 (±0.232)	0.112 (±0.260)	1.021 (±0.815)	0.156 (±0.404)	0.099 (0.280)
θ (×10^−3^)	0.077	0.080	2.434	0.133	0.084

Population abbreviations are defined in Table 1. π, nucleotide diversity; θ, nucleotide polymorphism. Numbers in parenthesis represent the standard deviation (sd) of the π values. Polymorphic genes: the genes contain one or more SNPs in each population.
