# Supplementary material for: Pronounced genetic differentiation and recent secondary contact in the mangrove tree Lumnitzera racemosa revealed by population genomic analyses
Source: Sci Rep. 2016 Jul 6;6:29486. doi: 10.1038/srep29486 (PMC4933931; doi:10.1038/srep29486)
Supplement: Supplementary Information [file srep29486-s1.pdf]

SUPPLEMENTARY INFORMATION

Pronounced genetic differentiation and recent secondary contact in the mangrove tree *Lumnitzera racemosa* revealed by population genomic analyses

Jianfang Li[1], Yuchen Yang[1], Qipian Chen[1], Lu Fang[1], Ziwen He[1], Wuxia Guo[1], Sitan Qiao[1], Zhengzhen Wang [1], Miaomiao Guo[1], Cairong Zhong[2],

Renchao Zhou[1]* and Suhua Shi[1]*

Supplementary Figure S1. Mismatch rates in each position of the reads (a) and the correlation between base quality and mismatch rate (b) in five populations of *Lumnitzera racemosa*. We trimmed the first five bases and the last seven bases of the mapped reads of QH, SY and KP population, and trimmed the first five bases and last ten bases of the mapped reads of JH and KD population. For five populations, we filtered the bases with quality scores lower than 22. Population abbreviations are defined in Table 1.

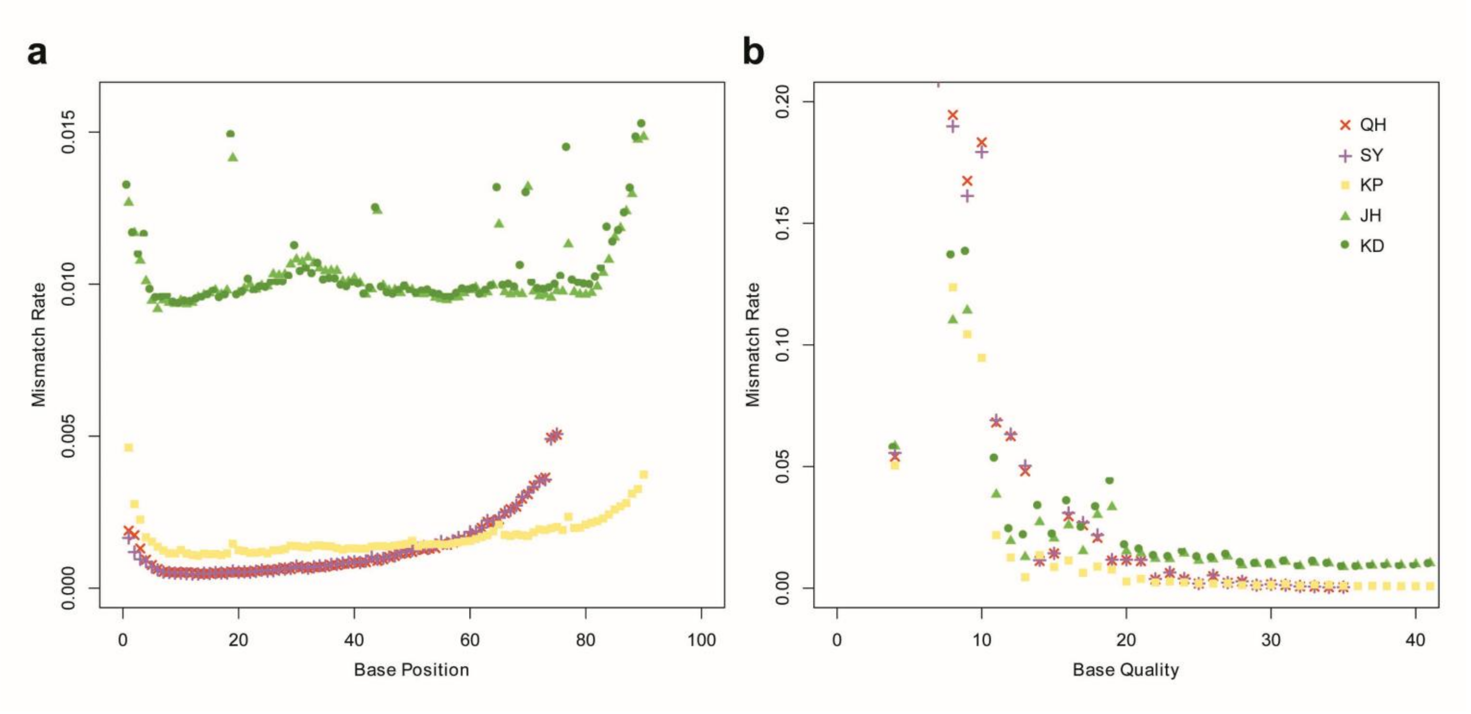

Supplementary Figure S2. Haplotype networks and distributions of 85 additional polymorphic gene segments in five populations. Each colour represents a population. Each circle represents a single haplotype. The size of the circle in each haplotype network is proportional to haplotype frequency. Each branch with more than one mutational step is labelled. Population abbreviations are defined in Table 1.

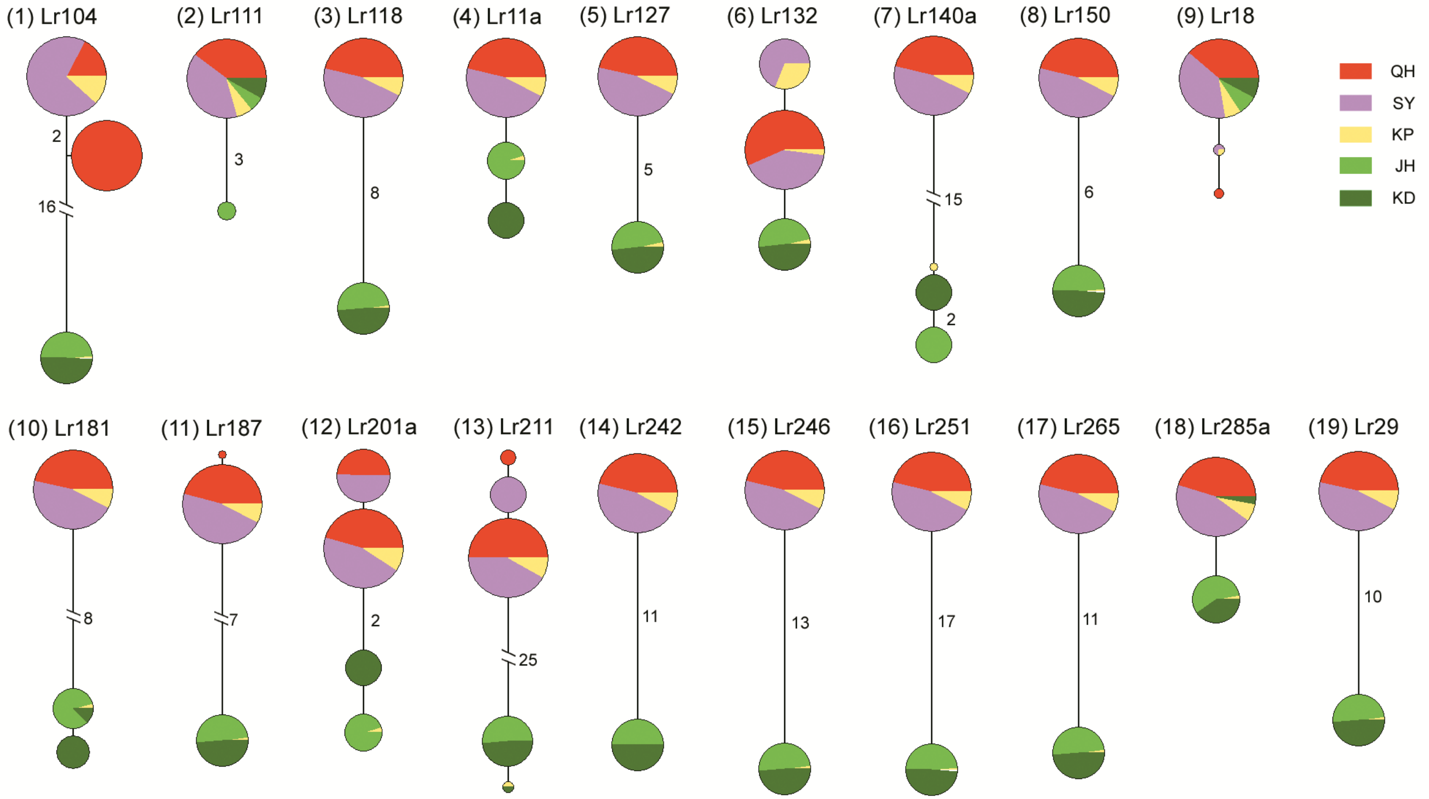

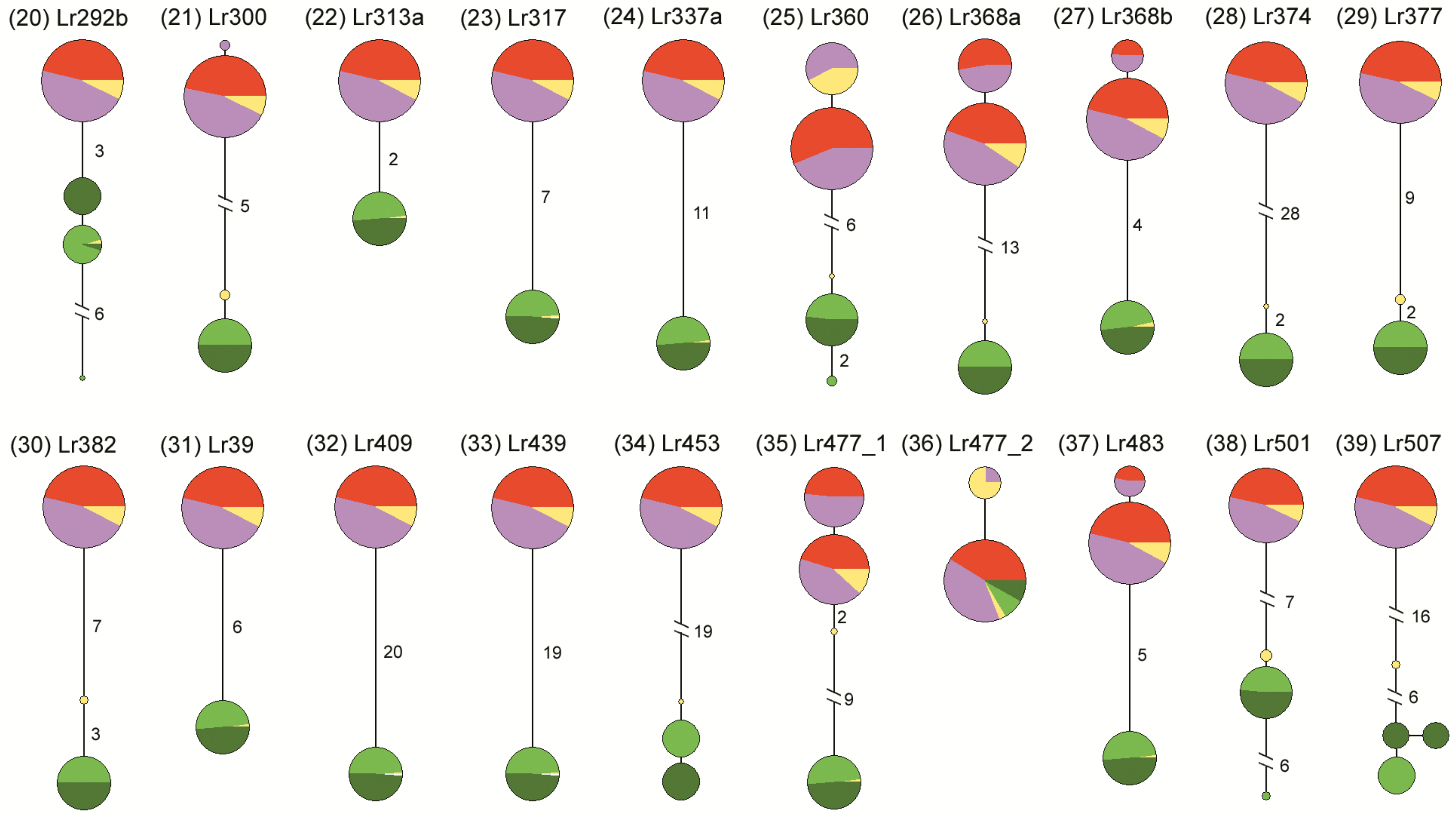

(20) Lr292b
3
6
(21) Lr300
5
(22) Lr313a
2
(23) Lr317
7
(24) Lr337a
11
(25) Lr360
6
2
(26) Lr368a
13
(27) Lr368b
4
(28) Lr374
28
2
(29) Lr377
9
2
(30) Lr382
7
3
(31) Lr39
6
(32) Lr409
20
(33) Lr439
19
(34) Lr453
19
(35) Lr477_1
2
9
(36) Lr477_2
(37) Lr483
5
(38) Lr501
7
6
(39) Lr507
16
6

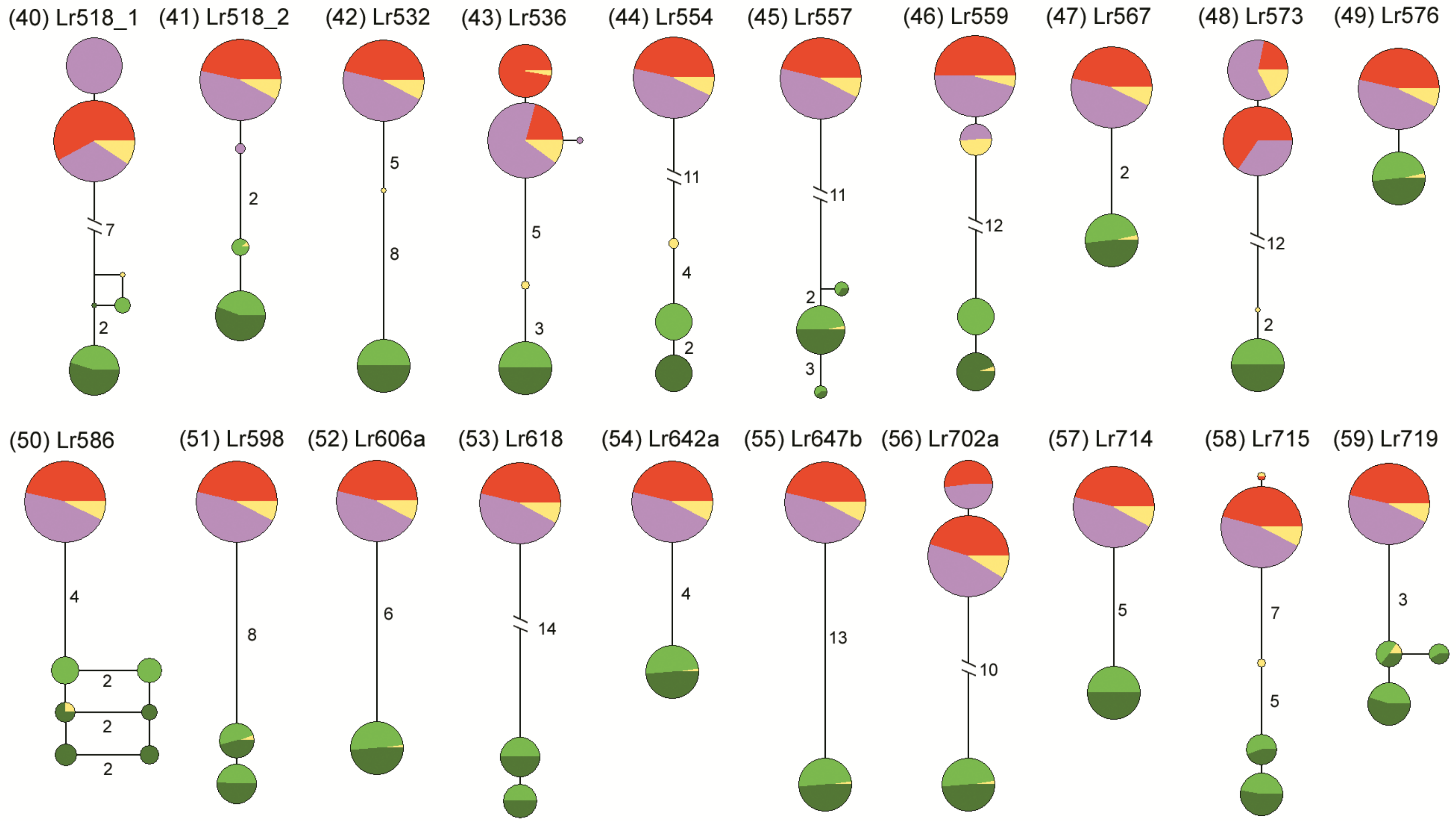

(40) Lr518_1
7
2
(41) Lr518_2
2
(42) Lr532
5
8
(43) Lr536
5
3
(44) Lr554
11
4
2
(45) Lr557
11
2
3
(46) Lr559
12
(47) Lr567
2
(48) Lr573
12
2
(49) Lr576
(50) Lr586
4
2
2
2
(51) Lr598
8
(52) Lr606a
6
(53) Lr618
14
(54) Lr642a
4
(55) Lr647b
13
(56) Lr702a
10
(57) Lr714
5
(58) Lr715
7
5
(59) Lr719
3

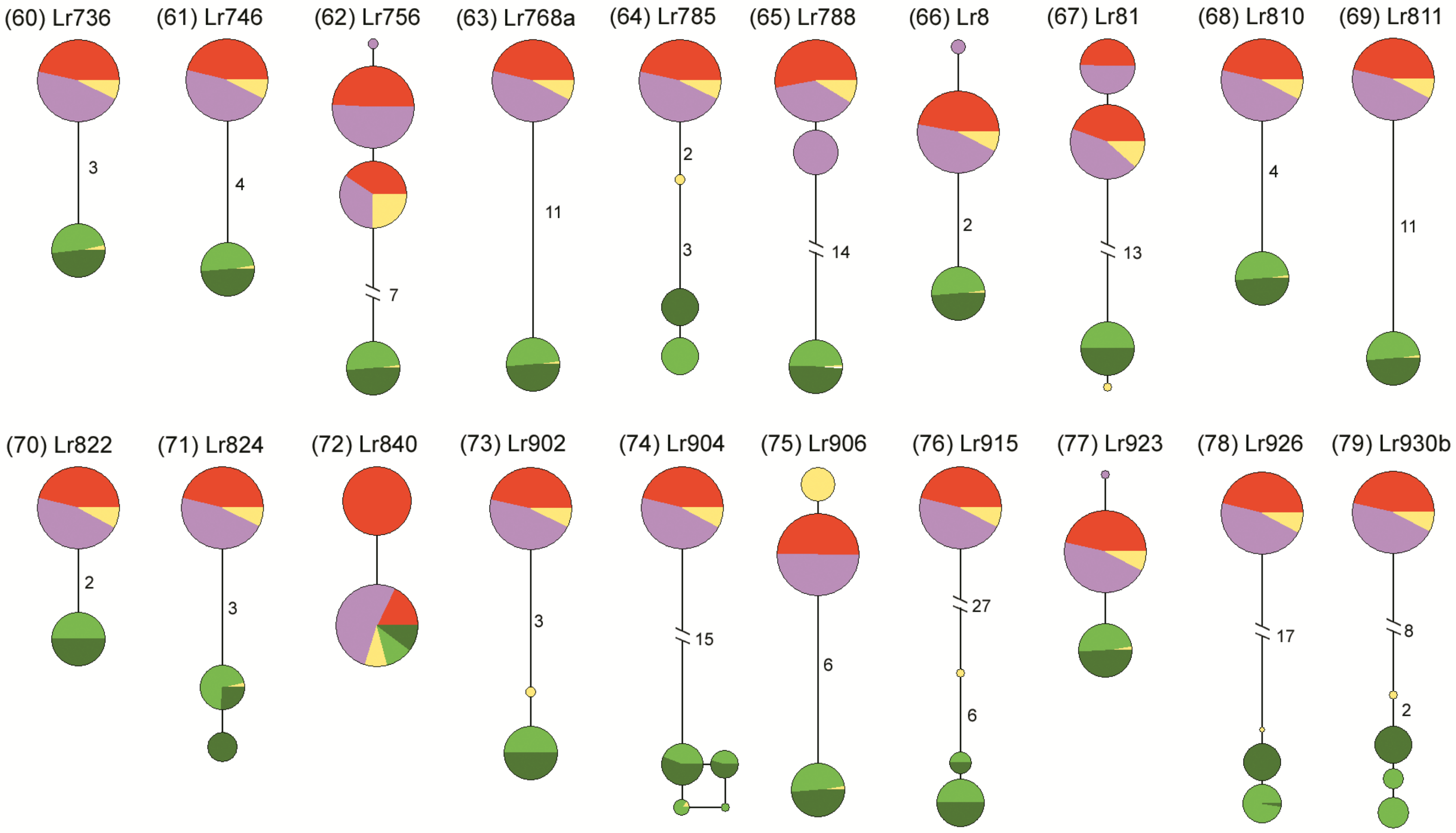
(60) Lr736
3
(61) Lr746
4
(62) Lr756
7
(63) Lr768a
11
(64) Lr785
2
3
(65) Lr788
14
(66) Lr8
2
(67) Lr81
13
(68) Lr810
4
(69) Lr811
11
(70) Lr822
2
(71) Lr824
3
(72) Lr840
(73) Lr902
3
(74) Lr904
15
(75) Lr906
6
(76) Lr915
27
6
(77) Lr923
(78) Lr926
17
(79) Lr930b
8
2

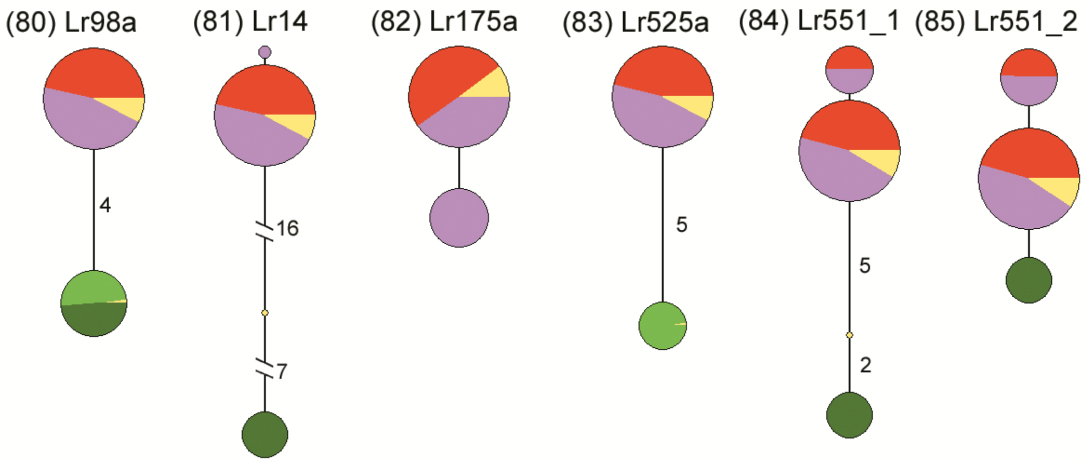
(80) Lr98a
(81) Lr14
(82) Lr175a
(83) Lr525a
(84) Lr551_1
(85) Lr551_2
4
16
7
5
5
2

Supplementary Figure S3. Plot of delta K values of 10 runs that assumed one to 10 clusters (K = 1 - 10).

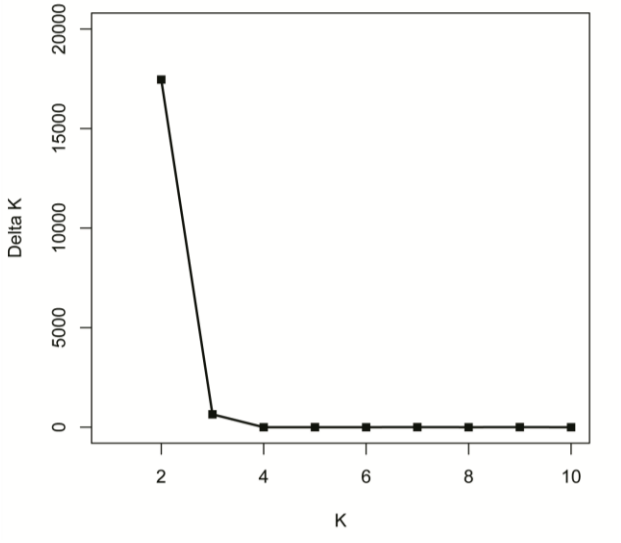

Supplementary Table S1. Population, sample size and nucleotide diversity at six nuclear genes of 17 populations of *Lumnitzera racemosa* from the Indo-West Pacific region. Alignment gaps were excluded for the calculation of nucleotide diversity (π) and polymorphism (θ). Population abbreviations are defined in Table 1.

Gene		GX	SM	SC	QH	SY	DW	QL	KP	YS	TN	KN	BL	TP	JH	KD	BT	RK	Total
Lr104	N	12	20	20	20	20	20	20	17	11	20	18	20	10	20	20	17	10	295
	S	0	3	0	3	0	4	4	19	3	4	19	19	0	0	0	0	0	22
	H	1	2	1	2	1	3	3	2	2	3	2	2	1	1	1	1	1	7
	Hd	0.000	0.050	0.000	0.409	0.000	0.555	0.512	0.214	0.312	0.631	0.056	0.142	0.000	0.000	0.000	0.000	0.000	0.692
	π ($\times10^{-3}$)	0.000	0.100	0.000	0.860	0.000	1.140	1.350	2.690	0.650	1.390	0.700	1.790	0.000	0.000	0.000	0.000	0.000	5.610
	θ ($\times10^{-3}$)	0.000	0.490	0.000	0.490	0.000	0.660	0.660	3.080	0.570	0.660	3.030	2.960	0.000	0.000	0.000	0.000	0.000	2.110
Lr265	N	12	20	20	20	20	20	20	17	11	20	18	20	10	20	20	17	10	295
	S	0	0	0	0	0	1	1	13	0	0	13	0	0	0	0	0	0	14
	H	1	1	1	1	1	2	2	2	1	1	2	1	1	1	1	1	1	4
	Hd	0.000	0.000	0.000	0.000	0.000	0.450	0.481	0.258	0.000	0.000	0.108	0.000	0.000	0.000	0.000	0.000	0.000	0.422
	π ($\times10^{-3}$)	0.000	0.000	0.000	0.000	0.000	0.000	0.000	3.370	0.000	0.000	1.410	0.000	0.000	0.000	0.000	0.000	0.000	5.320
	θ ($\times10^{-3}$)	0.000	0.000	0.000	0.000	0.000	0.000	0.000	3.190	0.000	0.000	3.150	0.000	0.000	0.000	0.000	0.000	0.000	2.050
Lr559	N	12	20	20	20	20	20	20	17	11	20	18	20	10	20	20	17	10	295
	S	0	1	1	1	1	0	0	14	14	14	13	1	14	0	0	0	0	14
	H	1	2	2	2	2	1	1	3	2	3	2	2	4	1	1	1	1	4
	Hd	0.000	0.508	0.262	0.097	0.142	0.000	0.000	0.629	0.173	0.344	0.056	0.050	0.437	0.000	0.000	0.000	0.000	0.698
	π ($\times10^{-3}$)	0.000	0.630	0.330	0.120	0.180	0.000	0.000	4.820	3.020	4.640	0.900	0.060	4.740	0.000	0.000	0.000	0.000	7.000
	θ ($\times10^{-3}$)	0.000	0.290	0.290	0.290	0.290	0.000	0.000	4.260	4.780	4.100	3.900	0.290	4.910	0.000	0.000	0.000	0.000	2.510
Lr598	N	12	20	20	20	20	20	20	17	11	20	17	20	8	19	20	17	10	291

	S	0	0	0	0	0	0	0	9	0	0	0	0	0	0	0	0	0	9
	H	1	1	1	1	1	1	1	2	1	1	1	1	1	1	1	1	1	2
	Hd	0.000	0.000	0.000	0.000	0.000	0.000	0.000	0.258	0.000	0.000	0.000	0.000	0.000	0.000	0.000	0.000	0.000	0.388
	π ($\times 10^{-3}$)	0.000	0.000	0.000	0.000	0.000	0.000	0.000	2.000	0.000	0.000	0.000	0.000	0.000	0.000	0.000	0.000	0.000	3.010
	θ ($\times 10^{-3}$)	0.000	0.000	0.000	0.000	0.000	0.000	0.000	1.890	0.000	0.000	0.000	0.000	0.000	0.000	0.000	0.000	0.000	1.120
Lr618	N	12	20	20	20	20	20	20	17	11	20	18	20	10	20	20	17	10	295
	S	0	0	0	0	0	0	0	15	0	0	15	0	15	1	0	0	1	19
	H	1	1	1	1	1	1	1	2	1	1	2	1	2	2	1	1	2	5
	Hd	0.000	0.000	0.000	0.000	0.000	0.000	0.000	0.214	0.000	0.000	0.056	0.000	0.100	0.097	0.000	0.000	0.442	0.515
	π ($\times 10^{-3}$)	0.000	0.000	0.000	0.000	0.000	0.000	0.000	3.320	0.000	0.000	0.860	0.000	1.550	0.110	0.000	0.000	0.490	6.410
	θ ($\times 10^{-3}$)	0.000	0.000	0.000	0.000	0.000	0.000	0.000	3.800	0.000	0.000	3.740	0.000	4.370	0.260	0.000	0.000	0.310	2.710
Lr81	N	12	20	20	20	20	20	20	17	11	19	18	20	9	19	20	17	10	292
	S	0	0	0	0	0	0	0	15	0	0	0	0	0	0	0	0	0	15
	H	1	1	1	1	1	1	1	2	1	1	1	1	1	1	1	1	1	2
	Hd	0.000	0.000	0.000	0.000	0.000	0.000	0.000	0.258	0.000	0.000	0.000	0.000	0.000	0.000	0.000	0.000	0.000	0.391
	π ($\times 10^{-3}$)	0.000	0.000	0.000	0.000	0.000	0.000	0.000	3.690	0.000	0.000	0.000	0.000	0.000	0.000	0.000	0.000	0.000	5.580
	θ ($\times 10^{-3}$)	0.000	0.000	0.000	0.000	0.000	0.000	0.000	3.490	0.000	0.000	0.000	0.000	0.000	0.000	0.000	0.000	0.000	2.060

Abbreviations: N, sample size; S, number of segregating (polymorphic) sites; H, number of haplotypes; Hd, haplotype diversity; π, nucleotide diversity; θ, nucleotide polymorphism.

Supplementary Table S2. Averages of pairwise F_{ST} values amongst five populations of *Lumnitzera racemosa* based on Illumina sequencing data. Numbers in parenthesis are standard deviation (sd) of the F_{ST} values. Population abbreviations are defined in Table 1.

	QH	SY	KP	JH	KD
QH					
SY	0.031 (±0.106)				
KP	0.065 (±0.138)	0.044 (±0.090)			
JH	0.923 (±0.217)	0.911 (±0.231)	0.847 (±0.196)		
KD	0.913 (±0.229)	0.901 (±0.241)	0.846 (±0.206)	0.156 (±0.334)	

Supplementary Table S3. Pairwise estimates of Kxy values at the gene Lr104 in 17 populations of *Lumnitzera racemosa*. Population abbreviations are defined in Table 1.

	GX	SM	SC	QH	SY	DW	QL	KP	YS	TN	KN	BL	TP	JH	KD	BT	RK
GX																	
SM	0.075																
SC	0.000	0.075															
QH	2.175	2.141	2.175														
SY	0.000	0.075	0.000	2.175													
DW	4.525	4.453	4.525	2.423	4.525												
QL	4.275	4.230	4.275	2.970	4.275	2.071											
KP	2.235	2.299	2.235	4.069	2.235	6.043	5.746										
YS	2.455	2.407	2.455	1.070	2.455	2.152	2.802	4.305									
TN	1.650	1.665	1.650	2.085	1.650	3.815	4.005	3.697	2.141								
KN	0.528	0.600	0.528	2.622	0.528	4.883	4.622	2.639	2.891	2.133							
BL	1.425	1.493	1.425	3.383	1.425	5.493	5.213	3.325	3.634	2.955	1.874						
TP	19.000	18.975	19.000	18.275	19.000	17.425	16.775	16.765	18.182	19.050	18.472	17.575					
JH	19.000	18.975	19.000	18.275	19.000	17.425	16.775	16.765	18.182	19.050	18.472	17.575	0.000				
KD	19.000	18.975	19.000	18.275	19.000	17.425	16.775	16.765	18.182	19.050	18.472	17.575	0.000	0.000			
BT	19.000	18.975	19.000	18.275	19.000	17.425	16.775	16.765	18.182	19.050	18.472	17.575	0.000	0.000	0.000		
RK	19.000	18.975	19.000	18.275	19.000	17.425	16.775	16.765	18.182	19.050	18.472	17.575	0.000	0.000	0.000	0.000	

Supplementary Table S4. Pairwise estimates of Kxy values at the gene Lr265 in 17 populations of *Lumnitzera racemosa*. Population abbreviations are defined in Table 1.

	GX	SM	SC	QH	SY	DW	QL	KP	YS	TN	KN	BL	TP	JH	KD	BT	RK
GX																	
SM	0.000																
SC	0.000	0.000															
QH	0.000	0.000	0.000														
SY	0.000	0.000	0.000	0.000													
DW	0.675	0.675	0.675	0.675	0.675												
QL	0.625	0.625	0.625	0.625	0.625	0.456											
KP	1.912	1.912	1.912	1.912	1.912	2.388	2.353										
YS	0.000	0.000	0.000	0.000	0.000	0.675	0.625	1.912									
TN	0.000	0.000	0.000	0.000	0.000	0.675	0.625	1.912	0.000								
KN	0.722	0.722	0.722	0.722	0.722	1.322	1.278	2.422	0.722	0.722							
BL	0.000	0.000	0.000	0.000	0.000	0.675	0.625	1.912	0.000	0.000	0.722						
TP	13.000	13.000	13.000	13.000	13.000	12.325	12.375	11.088	13.000	13.000	12.278	13.000					
JH	13.000	13.000	13.000	13.000	13.000	12.325	12.375	11.088	13.000	13.000	12.278	13.000	0.000				
KD	13.000	13.000	13.000	13.000	13.000	12.325	12.375	11.088	13.000	13.000	12.278	13.000	0.000	0.000			
BT	13.000	13.000	13.000	13.000	13.000	12.325	12.375	11.088	13.000	13.000	12.278	13.000	0.000	0.000	0.000		
RK	13.000	13.000	13.000	13.000	13.000	12.325	12.375	11.088	13.000	13.000	12.278	13.000	0.000	0.000	0.000	0.000	

Supplementary Table S5. Pairwise estimates of Kxy values at the gene Lr559 in 17 populations of *Lumnitzera racemosa*. Population abbreviations are defined in Table 1.

	GX	SM	SC	QH	SY	DW	QL	KP	YS	TN	KN	BL	TP	JH	KD	BT	RK
GX																	
SM	0.450																
SC	0.150	0.465															
QH	0.050	0.455	0.185														
SY	0.075	0.458	0.203	0.118													
DW	1.000	0.550	0.850	0.950	0.925												
QL	1.000	0.550	0.850	0.950	0.925	0.000											
KP	2.441	2.415	2.432	2.438	2.437	2.382	2.382										
YS	1.273	1.641	1.395	1.314	1.334	2.091	2.091	3.270									
TN	2.150	2.420	2.240	2.180	2.195	2.750	2.750	3.806	3.032								
KN	1.361	0.911	1.211	1.311	1.286	0.361	0.361	2.637	2.386	3.003							
BL	0.975	0.548	0.833	0.928	0.904	0.025	0.025	2.384	2.070	2.735	0.386						
TP	11.850	11.490	11.730	11.810	11.790	11.050	11.050	10.118	10.968	10.355	10.803	11.070					
JH	14.000	13.550	13.850	13.950	13.925	13.000	13.000	11.559	12.727	11.850	12.639	13.025	2.150				
KD	14.000	13.550	13.850	13.950	13.925	13.000	13.000	11.559	12.727	11.850	12.639	13.025	2.150	0.000			
BT	13.000	12.550	12.850	12.950	12.925	12.000	12.000	10.853	11.909	11.150	11.694	12.025	2.650	1.000	1.000		
RK	13.000	12.550	12.850	12.950	12.925	12.000	12.000	10.853	11.909	11.150	11.694	12.025	2.650	1.000	1.000	0.000	

Supplementary Table S6. Pairwise estimates of Kxy values at the gene Lr598 in 17 populations of *Lumnitzera racemosa*. Population abbreviations are defined in Table 1.

	GX	SM	SC	QH	SY	DW	QL	KP	YS	TN	KN	BL	TP	JH	KD	BT	RK
GX																	
SM	0.000																
SC	0.000	0.000															
QH	0.000	0.000	0.000														
SY	0.000	0.000	0.000	0.000													
DW	0.000	0.000	0.000	0.000	0.000												
QL	0.000	0.000	0.000	0.000	0.000	0.000											
KP	1.324	1.324	1.324	1.324	1.324	1.324	1.324										
YS	0.000	0.000	0.000	0.000	0.000	0.000	0.000	1.324									
TN	0.000	0.000	0.000	0.000	0.000	0.000	0.000	1.324	0.000								
KN	0.000	0.000	0.000	0.000	0.000	0.000	0.000	1.324	0.000	0.000							
BL	0.000	0.000	0.000	0.000	0.000	0.000	0.000	1.324	0.000	0.000	0.000						
TP	9.000	9.000	9.000	9.000	9.000	9.000	9.000	7.676	9.000	9.000	9.000	9.000					
JH	9.000	9.000	9.000	9.000	9.000	9.000	9.000	7.676	9.000	9.000	9.000	9.000	0.000				
KD	9.000	9.000	9.000	9.000	9.000	9.000	9.000	7.676	9.000	9.000	9.000	9.000	0.000	0.000			
BT	9.000	9.000	9.000	9.000	9.000	9.000	9.000	7.676	9.000	9.000	9.000	9.000	0.000	0.000	0.000		
RK	9.000	9.000	9.000	9.000	9.000	9.000	9.000	7.676	9.000	9.000	9.000	9.000	0.000	0.000	0.000	0.000	

Supplementary Table S7. Pairwise estimates of Kxy values at the gene Lr618 in 17 populations of *Lumnitzera racemosa*. Population abbreviations are defined in Table 1.

	GX	SM	SC	QH	SY	DW	QL	KP	YS	TN	KN	BL	TP	JH	KD	BT	RK
GX																	
SM	0.000																
SC	0.000	0.000															
QH	0.000	0.000	0.000														
SY	0.000	0.000	0.000	0.000													
DW	0.000	0.000	0.000	0.000	0.000												
QL	0.000	0.000	0.000	0.000	0.000	0.000											
KP	1.765	1.765	1.765	1.765	1.765	1.765	1.765										
YS	0.000	0.000	0.000	0.000	0.000	0.000	0.000	1.765									
TN	0.000	0.000	0.000	0.000	0.000	0.000	0.000	1.765	0.000								
KN	0.417	0.417	0.417	0.417	0.417	0.417	0.417	2.083	0.417	0.417							
BL	0.000	0.000	0.000	0.000	0.000	0.000	0.000	1.765	0.000	0.000	0.417						
TP	14.250	14.250	14.250	14.250	14.250	14.250	14.250	12.662	14.250	14.250	13.875	14.250					
JH	15.050	15.050	15.050	15.050	15.050	15.050	15.050	13.285	15.050	15.050	14.633	15.050	0.800				
KD	15.000	15.000	15.000	15.000	15.000	15.000	15.000	13.235	15.000	15.000	14.583	15.000	0.750	0.050			
BT	16.000	16.000	16.000	16.000	16.000	16.000	16.000	14.235	16.000	16.000	15.583	16.000	1.750	1.050	1.000		
RK	15.700	15.700	15.700	15.700	15.700	15.700	15.700	13.935	15.700	15.700	15.283	15.700	1.450	0.750	0.700	0.300	

Supplementary Table S8. Pairwise estimates of Kxy values at the gene Lr81 in 17 populations of *Lumnitzera racemosa*. Population abbreviations are defined in Table 1.

	GX	SM	SC	QH	SY	DW	QL	KP	YS	TN	KN	BL	TP	JH	KD	BT	RK
GX																	
SM	0.000																
SC	0.000	0.000															
QH	0.000	0.000	0.000														
SY	0.000	0.000	0.000	0.000													
DW	0.000	0.000	0.000	0.000	0.000												
QL	0.000	0.000	0.000	0.000	0.000	0.000											
KP	2.206	2.206	2.206	2.206	2.206	2.206	2.206										
YS	0.000	0.000	0.000	0.000	0.000	0.000	0.000	2.206									
TN	0.000	0.000	0.000	0.000	0.000	0.000	0.000	2.206	0.000								
KN	0.000	0.000	0.000	0.000	0.000	0.000	0.000	2.206	0.000	0.000							
BL	0.000	0.000	0.000	0.000	0.000	0.000	0.000	2.206	0.000	0.000	0.000						
TP	15.000	15.000	15.000	15.000	15.000	15.000	15.000	12.794	15.000	15.000	15.000	15.000					
JH	15.000	15.000	15.000	15.000	15.000	15.000	15.000	12.794	15.000	15.000	15.000	15.000	0.000				
KD	15.000	15.000	15.000	15.000	15.000	15.000	15.000	12.794	15.000	15.000	15.000	15.000	0.000	0.000			
BT	15.000	15.000	15.000	15.000	15.000	15.000	15.000	12.794	15.000	15.000	15.000	15.000	0.000	0.000	0.000		
RK	15.000	15.000	15.000	15.000	15.000	15.000	15.000	12.794	15.000	15.000	15.000	15.000	0.000	0.000	0.000	0.000	
